# High-Fat Diet Affects Ceramide Content, Disturbs Mitochondrial Redox Balance, and Induces Apoptosis in the Submandibular Glands of Mice

**DOI:** 10.3390/biom9120877

**Published:** 2019-12-15

**Authors:** Anna Zalewska, Mateusz Maciejczyk, Julita Szulimowska, Monika Imierska, Agnieszka Błachnio-Zabielska

**Affiliations:** 1 Experimental Dentistry Laboratory, Medical University in Bialystok, 15-437 Bialystok, Poland; 2 Department of Hygiene, Epidemiology and Ergonomics, Medical University in Bialystok, 15-437 Bialystok, Poland; 3 Department of Pediatric Dentistry, Medical University in Bialystok, 15-437 Bialystok, Poland; szulimowska.julita@gmail.com; 4 Department of Hygiene, Epidemiology and Metabolic Disorders, Medical University in Bialystok, 15-437 Bialystok, Poland; higienametz@umb.edu.pl (M.I.); agnieszka.blachnio@umb.edu.pl (A.B.-Z.)

**Keywords:** ceramide, insulin resistance, mitochondrial activity, redox homeostasis, antioxidants, salivary glands

## Abstract

This is the first study to investigate the relationship between ceramides, the mitochondrial respiratory system, oxidative stress, inflammation, and apoptosis in the submandibular gland mitochondria of mice with insulin resistance (IR). The experiment was conducted on 20 male C57BL/6 mice divided into two equal groups: animals fed a high-fat diet (HFD; 60 kcal% fat) and animals fed a standard diet (10 kcal% fat). We have shown that feeding mice HFD induces systemic IR. We noticed that HFD feeding was accompanied by a significant increase in ceramide production (C18 1Cer, C18 Cer, C22 Cer, C24 1Cer, C24 Cer), higher activity of pro-oxidant enzymes (NADPH oxidase and xanthine oxidase), and weakened functioning of mitochondrial complexes in the submandibular glands of IR mice. In this group, we also observed a decrease in catalase and peroxidase activities, glutathione concentration, redox status, increased concentration of protein (advanced glycation end products, advanced oxidation protein products) and lipid (malondialdehyde, lipid hydroperoxide) peroxidation products, and enhanced production of tumor necrosis factor alpha (TNFα) and interleukin 2 (IL-2) as well as pro-apoptotic Bax in the submandibular gland mitochondria. In summary, HFD impairs salivary redox homeostasis and is responsible for enhanced oxidative damage and apoptosis in the submandibular gland mitochondria. The accumulation of some ceramides could boost free radical formation by affecting pro-oxidant enzymes and the mitochondrial respiratory chain.

## 1. Introduction

In the modern world, particularly in the population of the so-called “western countries”, there is a growing pandemic of obesity. According to the current data of the World Health Organization, around 1.9 billion adults are overweight (body mass index, BMI > 25 kg/m^2^) and nearly 600 million are obese (BMI > 30 kg/m^2^). The basis of the increasing occurrence of obesity is a combination of genetic, environmental, and economic factors (easy access to cheap and high-calorie food) as well as evolutionary factors (sedentary lifestyle, little physical activity, low energy expenditure). Almost two-thirds of the American population are overweight and about 30% are clinically obese [1]. Obesity is a documented risk factor for the development of insulin resistance (IR), and consequently, type 2 diabetes [2].

In obesity and IR conditions, increased content of free fatty acids, diacylglycerols, triacylglycerols, and ceramides in the cell cytoplasm of various internal organs is observed [3,4,5,6]. Ceramides are composed of sphingosine connected by an amide bond with fatty acid. They vary in acyl-chain length ranging from C14:0 to C30:0. The ceramides most commonly found in nature are: C14:0-Cer, C16:0-Cer, C18:1-Cer, C18:0-Cer, C20:0-Cer, C22:0-Cer, C24:1-Cer, and C24:0-Cer. They form cell membranes and are precursors of more complex sphingolipids, such as sphingomyelin, ceramide-1 phosphate, and glycerosphingolipids. In addition to their structural function, ceramides play several roles in the cell, affecting its differentiation, proliferation, cellular arrest, and apoptosis; regulating the process of protein phosphorylation, as well as acting as a secondary transmitter and thus playing an important role in cell signaling [7]. However, it has been proven that intramuscular accumulation of ceramides, diacylglycerol, and long-chain acyl-CoAs participate in IR pathogenesis [8]. Interestingly, it appears that ceramide derivatives, mainly sphingosine-1-phosphate (S1P), influence cellular growth and function, and can thus be involved in the progression of IR-related organ complications [3,9]. The mechanisms by which ceramides induce IR are of a complex nature, connected with the induction of oxidative stress (OS). The available evidence indicates that ceramides activate NADPH oxidase (NOX) in coronary endothelium [10], induce disorders of the respiratory chain in rat liver mitochondria [11], enhance nitric oxide synthase activity (iNOS) in RAW264.7 cells [12], and inhibit the activity of anti-apoptotic Bcl-2 protein in LYas and LYar cells [13], which results in increased generation of oxygen and nitrogen free radicals as well as apoptosis in many mammalian cells [13,14].

Evidence implies that OS plays a key role in salivary gland function as well as disturbances of saliva composition in the course of IR [15,16,17,18,19]. In our previous works we demonstrated alterations of the antioxidant barrier [19], oxidative stress [16], and mitochondrial dysfunction [18] in salivary glands of rats with IR.

In the presented study, we aim to perform a detailed assessment of selected ceramides, mitochondrial redox balance as well as proteins responsible for apoptosis in the submandibular glands of IR mice, as these issues have never been explored before. It also appears highly probable that—as in the heart, liver, and skeletal muscle cells—there is a connection between the accumulation of ceramides and their derivatives inside salivary glands and disturbances of mitochondrial redox homeostasis and apoptosis. Therefore, the second aim of the study was to investigate the connection between the ceramides and mitochondrial respiratory system, selected mitochondrial antioxidants, and the activity of pro-oxidant enzymes as well as apoptosis markers in the submandibular glands of IR mice.

## 2. Materials and Methods

The study was approved by the Local Ethical Committee for Animal Experiments of the Medical University of Olsztyn, Poland (approval number 43/2016). The experiments were performed on male C57BL/6 mice (20 g) that were obtained from the Jackson Laboratory (Bar Harbor, Maine, USA). The animals were housed under standard conditions (21 ± 2 °C, 12-h shifts of the light–dark cycle) with free access to water and food pellets. The animals (*n* = 10) were randomly divided into two groups:

(1) Control group fed a control rodent diet ad libitum (Research Diets, New Brunswick, NJ, USA, D12450J).
(2) Group of animals fed a high-fat diet (HFD) ad libitum (Research Diets, New Brunswick, NJ, USA D12492).

In our experiment we used a high-fat diet (HFD) containing 60% fat, 20% protein, and 20% carbohydrates. The main source of fat was lard. The control diet contained 10% fat, 20% protein, and 70% carbohydrates. Table 1 contains information on the general composition of the individual diets. Table 2 shows the composition of fatty acids of each diet.

All the animals were fed the appropriate diet for 8 weeks. On the last day of the study, the mice were fasted for 6 h for blood glucose and insulin measurements. The mice were anesthetized by intraperitoneal injection of pentobarbital at a dose of 80 mg/kg body weight. Salivary glands were collected, frozen in liquid nitrogen, and then stored at −80 °C until assayed.

Based on our preliminary study (data not shown), no significant differences were found in the redox/sphingolipid biomarkers measured between the left and right salivary glands. Therefore, the right salivary glands were used for lipid analysis and the left salivary glands were used for mitochondrial activity determination. The salivary gland index was also calculated using the formula [20]:

salivary gland index = salivary gland weight/body weight × 100%(1)

### 2.1. Concentration of Plasma Insulin and Blood Glucose, Calculation of HOMA-IR

Blood glucose concentration was measured using the AccuChek glucometer. The plasma insulin concentration was determined by means of an ELISA insulin assay (Rat/Mouse Insulin, Millipore, Burlington, MA, USA). The insulin sensitivity was assessed using the homeostasis model assessment of insulin resistance (HOMA)-IR index using the formula [18]:

HOMA = fasting insulin (U/mL) × fasting glucose (mM)/22.5)(2)

### 2.2. Sphingolipids

Right submandibular glands of mice were used to assess the concentration of sphingolipids. The sphingolipid content was determined with the ultra-high-performance liquid chromatography-tandem mass spectrometry (UHPLC/MS/MS) approach according to Blachnio-Zabielska et al. [21] with minor modifications. In short, salivary gland samples (~20 mg) were pulverized and then homogenized in a solution composed of 0.25 M sucrose, 25 mM KCl, 50 mM Tris, and 0.5 mM EDTA, pH 7.4. Immediately afterwards, the internal standard (17C-sphingosine, 17C-S1P, C15-d7-Cer, C16:0-d7-Cer, C18:0-d7-Cer, C24:0-d7-Cer, C24:1-d7-Cer, d17:1/18:1-Cer, and d17:1/20:0-CerC16 ceramide-d7 (d18:1-d7/16:0) (Avanti Polar Lipids, Alabaster, AL, USA)) and the extraction mixture (isopropanol: water: ethyl acetate, 30:10:60; v/v/v) were added to each homogenate. The mixture was vortexed, sonicated, and then centrifuged for 10 min at 4000× *g* (Sorvall Legend RT). The supernatant was transferred to a new vial, and pellet was re-extracted. After centrifugation, the supernatants were combined together and evaporated under nitrogen. The dried sample was reconstituted in LC Solvent B (2 mM ammonium formate, 0.1% formic acid in methanol) for UHPLC/MS/MS analysis. Sphingolipids were analyzed with a Sciex QTRAP 6500 + triple quadrupole mass spectrometer (AB Sciex Germany GmbH, Darmstadt, Germany) using a positive ion electrospray ionization (ESI) source (except for S1P which was analyzed in the negative mode) with multiple reaction monitoring (MRM) against standard curves, constructed for each compound. The chromatographic separation was performed using Shimadzu ultra-performance liquid chromatography (UHPLC). The analytical column was a reverse-phase Zorbax SB-C8 column 2.1 × 150 mm, 1.8 μm (Agilent Technologies, Santa Clara, CA, USA). Chromatographic separation was conducted in binary gradient using 1 mM ammonium formate, 0.1% formic acid in water as solvent A, and 2 mM ammonium formate, 0.1% formic acid in methanol as solvent B at the flow rate of 0.4 mL/min.

### 2.3. Mitochondria Isolation

On the day of the collection of tissues, the left salivary glands were homogenized in ice-cold isolation buffer using Teflon-on-glass electric homogenizer (1:15, *w*/*v*) [3,4]. Mitochondrial isolation buffer (250 mmol/L sucrose, 5 mmol/L Tris-HCl, 2 mmol/L ethylene glycol bis(2-aminoethyl)tetraacetic acid (EGTA), pH 7.4) was prepared in ultra-pure water (Sigma-Aldrich, Germany) immediately before homogenization [3,4]. To prevent sample proteolysis, protease inhibitors were added (Complete Mini, Roche, Mannheim, Germany; 1 tablet/10 mL of the isolation buffer) [22]. Homogenates were centrifuged at 500*× g* (10 min, 4 °C) and the resulting supernatant was centrifuged twice at 8000*× g* (10 min, 4 °C). The mitochondria pellet was resuspended in ice-cold mitochondria isolation buffer and used immediately [18,22,23]. The purity of the mitochondrial fraction was assessed by Western blotting. We did not show the presence of glyceraldehyde 3-phosphate dehydrogenase (GAPDH, cytoplasmic marker) and histone H3 (nuclear marker) (data not shown).

### 2.4. Mitochondrial Antioxidants

The activity of superoxide dismutase (SOD, EC 1.15.1.1) was measured colorimetrically at a 340-nm wavelength by inhibiting the oxidation of epinephrine to adrenochrome [24]. It was assumed that one unit of SOD activity inhibited epinephrine oxidation by 50%.

The activity of catalase (CAT, EC 1.11.1.6) was determined colorimetrically at 340 nm wavelength by measuring hydrogen peroxide decomposition in the sample [25]. One unit of CAT activity was defined as an amount of the enzyme that degrades 1 μmol of hydrogen peroxide per minute.

The activity of salivary peroxidase (Px, EC 1.11.1.7) was assessed colorimetrically based on the reduction of 5,5’-dithiobis-(2-nitrobenzoic acid) (DTNB) to thio-nitrobenzoic acid at 412 nm wavelength [26].

The activity of glutathione reductase (GR, EC 1.6.4.2) was measured colorimetrically by monitoring the decrease in absorbance at 340 nm due to the oxidation of NADPH in the sample [27]. It was assumed that one unit of GR activity oxidizes 1 mmol of NADPH per minute.

The concentration of oxidized (GSSG) and reduced (GSH) glutathione were determined colorimetrically at a 412-nm wavelength based on the enzymatic reaction between NADPH, DTNB, and GR [28]. For the GSSG determination, samples were thawed and neutralized to pH 6–7 with 1 M chlorhydrol triethanolamine (TEA), and then incubated with 2-vinylpyridine (to inhibit glutathione oxidation). The concentration of GSH was calculated using the difference between the levels of total glutathione and GSSG. The oxidation/reduction (redox) ratio was calculated according to the formula [GSH]^2^/[GSSG].

### 2.5. Mitochondrial Oxidative Stress

The content of advanced glycation end products (AGE) was measured fluorimetrically at an excitation wavelength of 440 nm and an emission wavelength of 350 nm, and expressed in arbitrary fluorescence units (AFU)/mg protein [29].

The concentration of advanced oxidation protein products (AOPPs) was analyzed colorimetrically at 340 nm by measuring the total iodide ion-oxidizing capacity of the sample [29].

The concentration of malondialdehyde (MDA) was measured colorimetrically at a 535-nm wavelength using the thiobarbituric acid reactive substances (TBARS) method [30]. As a standard, 1,1,3,3-tetramethoxypropane was used.

The concentration of lipid hydroperoxides (LOOH) was measured bichromatically at 570/700 nm wavelength based on the oxidation of Fe^2+^ to Fe^3+^ by LOOHs, under acidic conditions [31]. H_2_O_2_ was used as a standard.

### 2.6. Mitochondrial ROS Production, Inflammation, and Apoptosis

The activity of NADPH oxidase (NOX, E.C. 1.6.3.1) was assessed using luminescence assay with lucigenin as an electron acceptor [32]. It was assumed that one unit of NOX activity is required to release 1 nmol of superoxide radical for a one minute.

The activity of xanthine oxidase (XO, E.C. 1.17.3.2.) was determined colorimetrically at a 290-nm wavelength by measuring the increase in absorbance of the released UA [33]. One unit of XO activity was defined as the amount of the enzyme required to release 1 μmol of UA per minute.

The rate of ROS formation was measured fluorimetrically at 488/525 nm. In this assay, 2,7-dichlorodihydrofluorescein diacetate (DCFH-DA) was used, which is de-esterified to 2,7-dichlorodihydrofluorescein (DCFH) by oxygen free radicals [34]. ROS production rate was calculated from the calibration curve for DCFH [35].

The concentrations of tumor necrosis factor α (TNF-α), interleukin 2 (IL-2), Bax, and Bcl-2 were estimated colorimetrically using commercial enzyme-linked immunosorbent assay (ELISA) kits according to the manufacturer’s instructions (Mouse Tumor necrosis factor, Mouse Interleukin-2, Mouse Apoptosis regulator BAXa, and Mouse Apoptosis regulator Bcl-2, all from EIAab, China). Briefly, specific antibodies suitably labeled with an enzyme were added to the antigen-coated plate. Depending on the amount of antigen, antibodies specifically bound to the antigen and unbound antibodies were eluted. After the addition of the substrate/chromophore, the enzyme catalyzed the reaction and the colored reaction product was measured colorimetrically at 405 nm.

The activity of caspase-3 (CAS-3, EC 3.4.22.56) was assayed colorimetrically at 405 nm wavelength using Ac-Asp-Glu-Val-Asp p-nitroanilide as a substrate [36].

### 2.7. Mitochondrial Activity

The activity of complex I (E.C. 1.6.5.3) was estimated colorimetrically at 600 nm wavelength based on 2,6-dichloroindophenol (DCIP) reduction by electrons accepted from coenzyme Q_1_ after the oxidation of NADH (reduced form of nicotinamide adenine dinucleotide) by complex I [37].

The activities of complex II (E.C. 1.3.5.1) and complex II+III (E.C. 1.10.2.2) were determined according to Rustin et al. [38] by measuring the activity of succinate-ubiquinone reductase and succinate-cytochrome c reductase, respectively.

The activity of complex IV (cytochrome c oxidase, COX) was analyzed colorimetrically at 550 nm by measuring the oxidation of reduced cytochrome c [39].

The activity of citrate synthase (CS) was measured colorimetrically at 412 nm wavelength in the reaction with 5-thio-2-nitrobenzoic acid, which is generated from 5,5′-dithiobis-2-nitrobenzoic acid during CS biosynthesis [40].

The production of mitochondrial hydrogen peroxide (H_2_O_2_) was determined fluorimetrically by measuring the increase in 530/590 nm wavelength due to the reaction of Amplex Red with hydrogen peroxide [41].

### 2.8. Mitochondrial Protein

The content of total mitochondrial protein was measured colorimetrically using the bicinchoninic acid (BCA) method with bovine serum albumin (BSA) as a standard (Thermo Scientific PIERCE BCA Protein Assay; Rockford, IL, USA). According to the manufacturer’s instructions, absorbance was measured at 562 nm.

### 2.9. Statistical Analysis

All results were standardized per mg of total protein. The statistical analysis was performed using GraphPad Prism 7 for MacOS (GraphPad Software, La Jolla, CA, USA). Normality of the distribution was confirmed by the Shapiro–Wilk test and therefore the unpaired Student’s *t*-test and Pearson correlation coefficient were used. The results were expressed as mean ± standard deviations (SD). The statistical significance was defined as *p* ≤ 0.05.

## 3. Results

### 3.1. General Characteristics

The average food intake was almost the same in both groups (data not shown). Body weight of mice fed the high-fat diet was significantly higher (by 31%) compared to the controls (*p* = 0.0009). The weight of the left and right salivary glands did not differ in both the control and HFD mice groups. However, the salivary gland index was significantly lower in the submandibular glands of HFD mice vs. control (both in the left and right salivary glands; *p* < 0.001, *p* < 0.001), while there was no statistically significant difference between the left and right salivary glands in both groups.

We noticed increased fasting glucose and insulin concentration in mice fed with high-fat diet compared to the animals fed with standard laboratory diet (↑ 71% *p* = 0.0008 and ↑ 93% *p* = 0.0009, respectively). Next we observed that high-fat diet led to IR expressed by elevated HOMA-IR index in animals fed with such diet (↑ 227% *p* = 0.0001) (Table 3).

### 3.2. Effect of High-Fat Diet on Ceramide Contents as Well as S1P, Sphinganine, and Sphingosine Concentration

**Figure 1:**
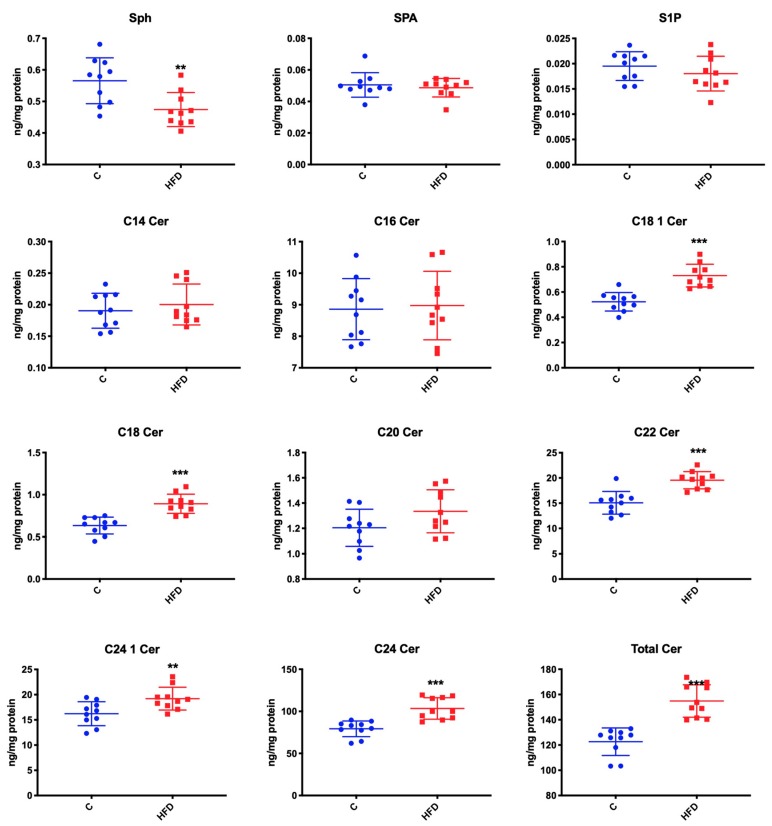
Effect of high-fat diet on ceramide content, S1P and SPA concentration and Sph activity. C—control, HFD—high-fat diet mice, Sph—sphingosine, SPA—sphinganine, S1P—sphingosine-1-phosphate, Cer—ceramide ** *p* < 0.005, *** *p* < 0.0005.

Compared to the control group, mice fed on high-fat diet were characterized by a significant increase in the concentration of C18:1-Cer (↑ 40% *p* < 0.001), C18 Cer (↑ 41% *p* < 0.001), C22 Cer (↑ 29% *p* < 0.001), C24:1 Cer (↑ 18% *p* = 0.01), C24 Cer (↑ 30% *p* < 0.001), and total Cer (↑ 26% *p* < 0.001) (Figure 1). However, the group receiving high-fat diet demonstrated a significant reduction in Sph concentration (↓ 18% *p* = 0.005). We also found that high-fat feeding did not change SPA, S1P, C14 Cer, C16 Cer, or C20 Cer concentrations compared to the controls (Figure 1). 

### 3.3. Effect of High-Fat Diet on Mitochondrial Antioxidants and Redox Ratio

**Figure 2:**
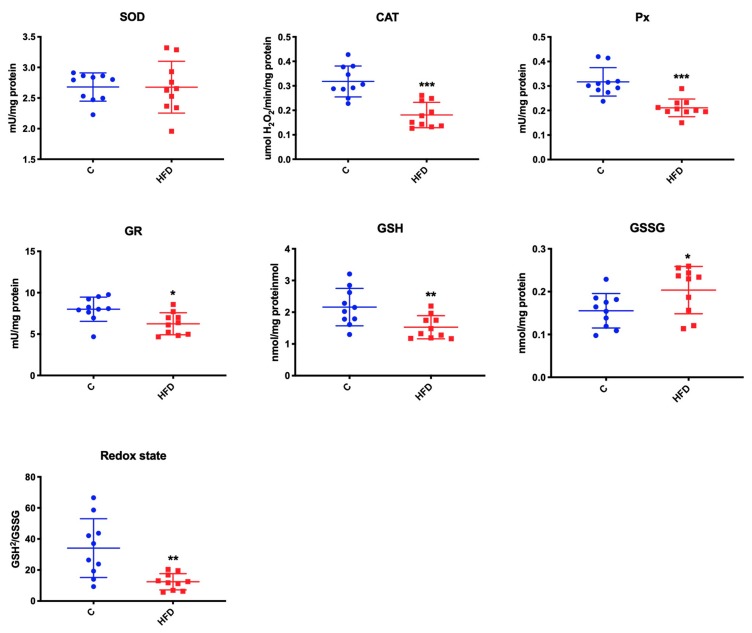
Effect of high-fat diet on mitochondrial antioxidants and redox ratio. C—control, HFD—high-fat diet mice, SOD—superoxide dismutase, CAT—catalase, Px—peroxidase, GR—glutathione reductase, GSH—reduced glutathione, GSSG—oxidized glutathione, [GSH]^2^/[GSSG]—redox ratio, * *p* < 0.05, ** *p* < 0.005, *** *p* < 0.0005.

We noticed that high-fat feeding had no effect only on SOD activity. In comparison with the control group, high-fat diet feeding caused a significant reduction of CAT, Px, and GR activity (↓ 44% *p* < 0.001, ↓ 34% *p* < 0.001, ↓ 22% *p* = 0.01, respectively) as well as GSH concentration (↓ 29% *p* = 0.01) (Figure 2). GSSG concentration was higher, while redox ratio was lower in the submandibular glands of mice receiving high-fat diet compared to the controls (↑ 33% *p* = 0.04 and ↓ 63% *p* = 0.003, respectively).

### 3.4. Effect of High-Fat Diet on Mitochondrial Oxidative Stress

**Figure 3:**
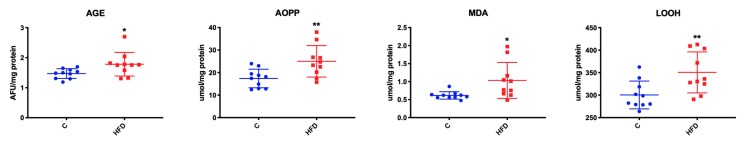
Effect of high-fat diet on mitochondrial oxidative stress. C—control, HFD—high-fat diet mice, AGE—advanced glycation end product, AOPP—advanced oxidation protein product, MDA—malondialdehyde, LOOH—lipid hydroperoxides, * *p* < 0.05, ** *p* < 0.005.

Compared to the controls, mice fed with high-fat diet were characterized by significant elevation in the concentration of AGE (↑ 21% *p* = 0.03), AOPP (↑ 44% *p* = 0.008), LOOH (↑ 17% *p* = 0.01), and MDA (↑ 66% *p* = 0.02) (Figure 3).

### 3.5. Effect of High-Fat Diet on Mitochondrial Respiratory Complexes and CS Activity as well as Mitochondrial H_2_O_2_ Production

**Figure 4:**
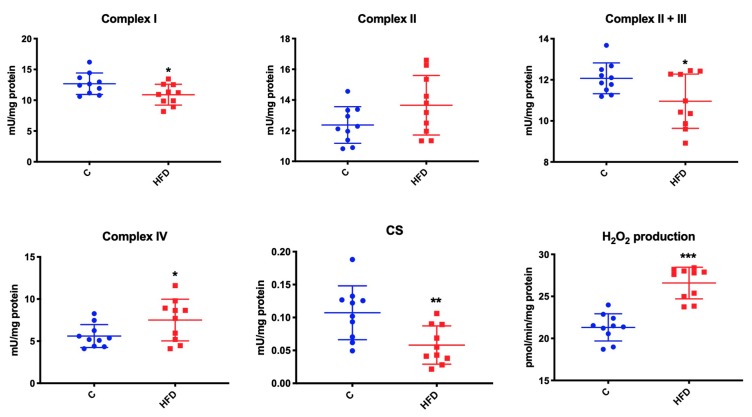
Effect of high-fat diet on mitochondrial respiratory complexes and enzyme activity. C—control, HFD—high-fat diet mice, CS—citrate synthase, * *p* < 0.05, ** *p* < 0.005, *** *p* < 0.0005.

High-fat feeding had no affect only on the activity of complex II. Compared to the control group, mice fed with high-fat diet were characterized by significant reduction in complex I, II + III, and CS activity (↓ 15% *p* = 0.03, ↓ 10% *p* = 0.03, ↓ 50% *p* = 0.006, respectively), whereby we observed a significant increase in the activity of complex IV (↑ 25% *p* = 0.05) and hydrogen peroxide production (↑ 20% *p* < 0.001) in the study group compared to the controls (Figure 4).

### 3.6. Effect of High-Fat Diet on Mitochondrial ROS Production, Inflammation, and Apoptosis

**Figure 5:**
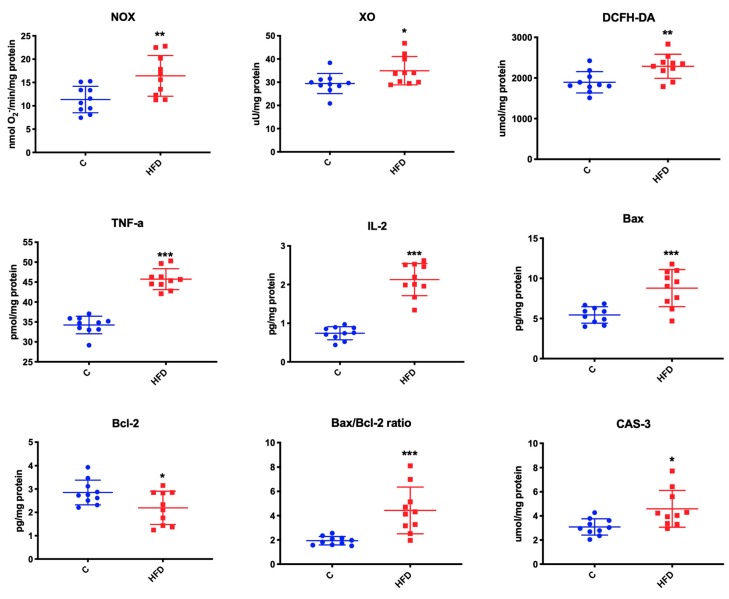
Effect of high-fat diet on mitochondrial ROS production, inflammation and apoptosis. C—control, HFD—high-fat diet mice, NOX—NADPH oxidase, XO—xanthine oxidase, DCFH-DA—rate of ROS formation, TNFα- tumor necrosis factor α, IL-2—interleukin 2, B cell lymphoma-2 family, Bax—pro-apoptic protein, Bcl-2—anti-apoptic protein, CAS-3—caspase 3, * *p* < 0.05, ** *p* < 0.005, *** *p* < 0.0005.

We observed that high-fat feeding increased the activity of mitochondrial NOX (↑ 31% *p* = 0.006), and XO (↑ 16% *p* = 0.03) compared to the mice fed normal chow (Figure 5).

Mice fed on high-fat diet demonstrated significantly higher TNF-α, IL-2, and Bax concentration as well as Bax/Bcl2 ratio and CAS-3 activity (↑ 25% *p* < 0.001; ↑ 187% *p* < 0.001; ↑ 38% *p* < 0.001, ↑ 131% *p* < 0.001, ↑ 33% *p* = 0.01, respectively). We also observed that the concentration of anti-apoptotic protein Bcl-2 was considerably lower in the group of mice fed a high-fat diet than in the control group (↓ 23% *p* = 0.03) (Figure 5).

### 3.7. Correlations

We demonstrated a positive correlation between the activity of complex I and the concentration of C18 Cer as well as C22 Cer (r = 0.851, *p* = 0.002), and a negative correlation between CS activity and C24 Cer concentration in the submandibular glands of IR mice (r = −0.853, *p* = 0.002). We observed a positive correlation between TNF-α concentration and total ceramide (r = 0.793, *p* = 0.006).

We obtained a negative correlation between mitochondrial AOPP concentration and the concentration of GSH (r = −0.665, *p* = 0.036) in the mitochondria of the submandibular glands in HFD mice.

There was a negative correlation between the activity of caspase 3 and the concentration of mitochondrial proteins (r = −0.735, *p* = 0.0155) and the activity of peroxidase (r = −0.816, *p* = 0.004). We also found a positive correlation between the content of C18 Cer and the activity of caspase 3 (r = 0.424, *p* = 0.05), as well as a negative correlation between the concentration of C24 Cer and Bcl-2 protein (r = −0.831, *p* = 0.003). Moreover, we noted a positive correlation between S1P and mitochondrial activity of NOX (r = 0.936, *p* < 0.0001).

## 4. Discussion

To our knowledge, the analysis of the ceramides, mitochondrial redox balance as well as proteins associated with apoptosis of the submandibular gland mitochondria has not been previously described in IR conditions.

In the presented experiment, we used a model of IR induced by a high-fat diet. After 8 weeks of the study we observed a 30% increase in body weight, a nearly 100% increase in insulin content, and a 70% increase in the concentration of glucose. We also noted that HFD led to IR, as confirmed by 200% increase in HOMA-IR, which is consistent with our results as well as those of other researchers [6,16,19,42,43].

Moreover, our study confirms the previous results indicating weakened functioning of mitochondrial complexes and increased mitochondrial production of ROS in the salivary glands of rats with HFD-induced IR [18]. However, only the submandibular gland of the mice was used in the study since the volume of the material obtained from the parotid glands would be insufficient to perform all the assays.

Excess fatty acids are stored in adipocytes and are used as a source of energy during fasting. It has been evidenced that, after exceeding the buffer capacity of adipose tissue—as in the high-fat diet conditions—lipids accumulate mainly in such tissues as liver, heart, pancreas, and skeletal muscles, causing dysfunction of the occupied organ known as lipotoxicity [44]. Lipid accumulation has also been observed in salivary glands. Matczuk et al. [6] showed that IR induced by chronic feeding with high-fat diet results in altered lipid fractions of salivary glands. These changes were expressed differently depending on the salivary gland type. The authors believe that the reduction of phospholipid concentration in salivary glands may be associated with their atrophy and reduced saliva secretion in the course of IR. However, the accumulation of the triacylglycerol fraction in submandibular glands is most probably a reflection of systemic disorders in lipid metabolism, observed in chronic implementation of high-fat diet [6].

We noticed that IR induced by a high-fat diet is accompanied by significant increase in the concentration of more toxic C 18 1Cer, C18 Cer, C22 Cer, C24 1Cer, and C24 Cer in cells of HFD-fed mice compared to the controls, which is consistent with the results of Veret et al. [45]. The authors have also demonstrated that the increase in these ceramides results in intensified apoptosis of pancreatic β cells [45]. The available evidence has indicated that ceramide induces apoptosis by: increasing mitochondrial cell membrane permeability by cytochrome c, activation of Bax and the reduction of Bcl-2 mRNA expression [45,46]. All of this leads to the release of cytochrome c as well as protein apoptotic protease activating factor 1 to cytoplasm, followed by the activation of caspase 9 and 3 that propagates apoptosis signal [46]. In our study we found a negative correlation between C24 Cer content and the concentration of Bcl-2 protein. We also noted a positive correlation between S1P and the mitochondrial activity of NOX. As described earlier [10,47], ceramide-dependent NOX activation increases the production of ROS and apoptosis, particularly in case of an inefficient antioxidant barrier—the situation was also observed in the presented results (no changes in SOD activity, ↓ Px, ↓ CAT, ↓ GSH). Moreover, it should be mentioned that mitochondria mainly contain the isoform NOX4 that predominantly produces H_2_O_2_ [48,49]. H_2_O_2_ is not a free radical, although it could trigger signal transduction pathways by nuclear factor κB and elevate the release of pro-inflammatory cytokines, which we observed as an increase in TNF-α concentration. In addition to the above-mentioned correlations, we observed increased Bax concentration, decreased Bcl-2, and boosted caspase 3 activity, which clearly indicates intensified apoptosis of submandibular glands of HFD-fed mice. A negative correlation between the activity of caspase 3, concentration of mitochondrial proteins, and peroxidase activity may suggest that apoptotic death of submandibular gland cells is so severe that leads to impairment of their function, as observed earlier by de la Cal et al. [50]. It is noteworthy that peroxidase is the only protein produced exclusively in salivary glands and is a determinant of their secretory function [19].

The previous in vitro studies showed that ceramide is able to modify electron transport in the respiratory chain and induce the production of ROS isolated in the heart and liver mitochondria [51,52,53]. Based on the obtained results, we can assume that ceramides may play a role in the pathogenesis of mitochondrial dysfunction and increased ROS generation provoked by HFD also in the submandibular glands of mice. We demonstrated that decreased catalytic activity of complex I is intensified with the increase in C18 Cer and C22 Cer concentration, and CS activity is reduced along with the increase of C24 Cer concentration in the subabdominal glands of IR mice. There are several hypotheses to explain the influence of ceramide on the activity of the respiratory chain. Ceramide may disturb the hydrophobicity of mitochondrial membranes, which results in the dysfunction of protein–lipid bilayer, entailing the disturbed structure and functioning of mitochondrial complexes [54]. Kota et al. [55] demonstrated that ceramide may act as an allosteric effector by binding with individual complexes of the respiratory chain and thus modifying their activity. However, further studies are required to mechanistically explain how ceramides affect salivary mitochondrial homeostasis in insulin resistance conditions.

It is worth noting that Bcl-2 protein prevents excessive ROS production as well as increasing the GSH pool and redistributing it [13]. With decreased Bcl-2 concentration, we observed a 33% decrease in GSH content, a 22% decrease in GR activity, a 44% decrease in CAT activity, and a 34% reduction in Px activity in isolated mitochondria of IR mice. Moreover, considering that GR reduces GSSG to GSH, the low ratio of intra-mitochondrial GSH to GSSG (↓ 63%) is not surprising. On the other hand, assuming that mitochondrial GSH is a result of the activity of ATP-dependent carrier that translocates cytoplasmic GSH to mitochondria [56], it may be presumed that there are disturbances of this transport in the cells of submandibular glands of IR mice. Because reduced gluathione is the only line of defense capable of metabolism of peroxides produced in the mitochondrial chain through the GSH redox cycle, and catalase and peroxidase play critical roles in redox signaling by cleavage of H_2_O_2_, the observed increase in H_2_O_2_ concentration in mitochondria is not surprising. It was demonstrated that at increased concentration of H_2_O_2_ the process of lipid peroxidation in liver mitochondria is intensified [11], which seems to occur also in the mitochondria of submandibular glands of IR mice (17% ↑ LOOH and 66% ↑ MDA). The negative correlation between mitochondrial AOPP concentration and the concentration of GSH in the mitochondria of the submandibular glands of HFD-fed mice suggests that increased oxidative modifications of mitochondrial proteins (44% ↑ AOPP) is the result of mitochondrial glutathione deficiency (↓ GSH concentration). The mitochondrial antioxidant barrier of submandibular glands of IR mice may be weakened by oxidative modification of polypeptide chains of enzymatic proteins or exhaustion of antioxidant reserve under the conditions of excess free radicals (20% ↑ H_2_O_2_ in mitochondria).

It should be underlined that in our research we measured the content of ceramides containing various fatty acids, differing in the length of the acyl chain and the number of double bonds. The ceramides we analyzed are those that are most commonly found in nature. Of course, different tissues represent a different ceramide profile. Since the metabolism of sphingolipids in the salivary glands is not well understand, we aimed to study the content of all ceramides that occur in nature in the largest amount.

Analyzing the possible paths of ceramide synthesis it can be concluded that an increase in total Cer concentration, accompanied by a decrease in Sph content and no changes in S1P and SPA concentration, suggests that the production of ceramide in the submandibular glands of IR mice occurs mainly by sphingomyelin hydrolysis [57,58]. This may be additionally confirmed by a positive correlation between TNF-α concentration and total ceramide. It has been evidenced that TNF-α could be activated by both neutral and acid sphingomielinases that cut the membrane sphingomyelin and consequently form ceramide, which is accompanied by ROS generation [59,60]. However, these hypotheses require thorough verification.

## 5. Conclusions

(1) A high-fat diet regimen increases the salivary gland ceramide composition.
(2) A high-fat diet intensifies oxidative damage to proteins and lipids and results in inflammation and apoptosis of submandibular gland mitochondria in mice.
(3) The accumulation of some ceramides appears to boost ROS production by affecting NOX activity and complexes I, II + III, and IV in the submandibular gland mitochondria of mice fed a high-fat diet.

## Figures and Tables

**Table 1:** General composition of the control and high-fat diet (HFD).

	Ingredient	Control Diet (g)	HFD (g)
Protein	Casein, lactic, 30 mesh	200.00	200.00
	Cystine, L	3.00	3.00
Carbohydrate	Starch, corn	506.20	0.00
	Lodex 10	125.00	125.00
	Sucrose, fine granulated	72.80	72.80
Fiber	Solka floc, FCC200	50.00	50.00
Fat	Soybean oil, USP	25.00	25.00
	Lard	20.00	245.00
Mineral	S10026B	50.00	50.00
Vitamin	Choline bitartrate	2.00	2.00
	V10001C	1.00	1.00

**Table 2:** Total fatty acid composition of purified diets.

	Control Diet (mg/g)	HFD (mg/g)
C14:0, Myristic	2.20 ± 0.20	4.43 ± 0.24 *
C16:0, Palmitic	9.21 ± 1.56	58.25 ± 1.10 *
C16:1, Palmitoleic	0.55 ± 0.12	4.84 ± 0.09 *
C18:0, Stearic	3.55 ± 0.60	27.73 ± 0.43 *
C18:1, Oleic	13.50 ± 2.24	90.83 ± 5.04 *
C18:2, Linoleic	12.42 ± 2.06	50.36 ± 1.04 *
C18:3, Linolenic	0.52 ± 0.09	2.91 ± 0.06 *
C20:0, Arachidic	0.11 ± 0.02	0.41 ± 0.01 *
C20:4, Arachidonic	0.05 ± 0.00	0.43 ± 0.01 *
C20:5, EPA	0.00 ± 0.00	0.03 ± 0.01 *
C22:0, Behenic	0.03 ± 0.00	0.07 ± 0.01 *
C22:6, DHA	0.01 ± 0.01	0.06 ± 0.01 *
C24:0, Lignoceric	0.04 ± 0.00	0.05 ± 0.00
C24:1 Selacholeic	0.00 ± 0.00	0.03 ± 0.00 *
Total	42.19 ± 6.85	240.43 ± 7.93 *

Values are expressed as mean ± SD. * *p* < 0.05 vs. control group.

**Table 3:** General characteristics of the control (C) and high-fat diet (HFD) mice.

	C (*n* = 10)	HFD (*n* = 10)	*p*
Body weight (g)	28.75 ± 0.51	37.88 ± 0.63	<0.001
Left salivary gland weight (g)	0.092 ± 0.01	0.097 ± 0.01	NS
Right salivary gland weight (g)	0.091 ± 0.002	0.093 ± 0.003	NS
Left salivary gland index (%)	0.32 ± 0.03	0.25 ± 0.03	NS
Right salivary gland index (%)	0.32 ± 0.03	0.26 ± 0.03	NS
Glucose (mg/dL)	101.5 ± 3.79	173.1 ± 8.77	<0.001
Insulin (ng/mL)	0.83 ± 0.02	1.6 ± 0.04	<0.001
HOMA-IR	3.34 ± 0.17	10.93 ± 0.57	<0.001
Total protein (μg/mL)	4004.5 ± 229	3671 ± 483	0.04

HOMA-IR—homeostasis model assessment of insulin resistance, NS—no significance. salivary gland index = salivary gland weight/body weight × 100%.
